# Sperm Accumulation Induced by the Female Reproductive Fluid: Putative Evidence of Chemoattraction Using a New Tool

**DOI:** 10.3390/cells10092472

**Published:** 2021-09-18

**Authors:** Alessandro Devigili, Silvia Cattelan, Clelia Gasparini

**Affiliations:** Department of Biology, University of Padova, via Ugo Bassi 58/B, 35131 Padova, Italy; silvia.cattelan@unipd.it (S.C.); clelia.gasparini@unipd.it (C.G.)

**Keywords:** ejaculate selection, postcopulatory selection, IVF, gametes, model organism, cryptic female choice, ovarian fluid, microfluidic

## Abstract

There is considerable evidence that female reproductive fluid (FRF) interacts intimately with sperm, affecting several sperm traits, including sperm motility and longevity, and ultimately fertilization success. One of the first documented interactions between FRF and sperm is the ability of FRF to attract and guide sperm towards the eggs. However, most of the evidence of FRF’s chemoattraction proprieties comes from a limited number of taxa, specifically mammals and invertebrate broadcasting spawners. In other species, small FRF volumes and/or short sperm longevity often impose methodological difficulties resulting in this gap in chemoattraction studies in non-model species. One of the outcomes of sperm chemotaxis is sperm accumulation towards high chemoattractant concentrations, which can be easily quantified by measuring sperm concentration. Here, we tested sperm accumulation towards FRF in the zebrafish, *Danio rerio*, using an ad hoc developed, 3D printed, device (‘sperm selection chamber’). This easy-to-use tool allows to select and collect the sperm that swim towards a chemical gradient, and accumulate in a chemoattractant-filled well thus providing putative evidence for chemoattraction. We found that sperm accumulate in FRF in zebrafish. We also found that none of the sperm quality traits we measured (sperm swimming velocity and trajectory, sperm motility, and longevity) were correlated with this response. Together with the 3D printable project, we provide a detailed protocol for using the selection chamber. The chamber is optimized for the zebrafish, but it can be easily adapted for other species. Our device lays the foundation for a standardized way to measure sperm accumulation and in general chemoattraction, stimulating future research aimed at understanding the role and the mechanisms of sperm chemoattraction by FRF.

## 1. Introduction

Female reproductive fluids (FRF)—i.e., the fluid surrounding the eggs, follicular or ovarian fluids, or other medium arising from females that interacts with sperm [1]—are among the female secretions that more intimately come into contact with male gametes. It is thus not surprising that such fluids can profoundly affect the physiology, behavior, and functionality of spermatozoa. Evidence of sperm–FRF interactions is widespread in animals, and an increasing number of studies have documented the effects of these interactions (reviewed in [1,2]).

Chemoattraction, the capacity to attract spermatozoa along a chemical gradient, represents a classical example of FRF–ejaculate interaction. The ability of sperm to respond to FRF and eggs has been long recognized [3] in both external (e.g., [4,5]) and internal fertilizing species [6], including humans [7,8]. Interestingly, chemoattraction by FRF has been directly associated with improved fertilization success (e.g., [9]) and embryo viability both when sperm were tested using FRF from different partners (e.g., [10]) and when sperm were tested using FRF from only one partner [11]. In the latter, in vitro fertilizations (IVF) performed using boar sperm selected based on their chemotactic response towards FRF led to an improved early embryo development compared with IVF performed with non-selected sperm [11]. Understanding the mechanisms and consequences of sperm chemoattraction towards FRF is thus clearly crucial to fully understand the processes that govern fertilization and lead to successful reproduction.

Assessing chemoattraction by FRF is technically equivalent to assessing chemoattractions using a specific chemoattractant, as, for example, ‘Resact’ in the sea urchins *Arbacia punctulata* [12], ‘Asterosap’ in the starfish *Asterias amurensis* [13], ‘SAAF’ in the ascidian *Ciona intestinalis* [14], or ‘Allurin’ in the frog *Crinia Georgiana* [15]. In mammals, a series of molecules have been tested for sperm chemoattraction, with the most common being progesterone [16]. Many technological improvements in testing and recreating chemoattraction in vitro have been made, most of which are functional to clinical activity and specifically focused on mammals—mice, rats, and humans, e.g., [17,18,19,20]. However, much research on reproductive biology and sexual selection is done in non-mammalian species, which have ejaculates that may exhibit drastically different characteristics, such as small volumes and sperm typically showing far shorter longevity compared with mammals or invertebrate broadcasters. These differences make it difficult to use techniques developed for mammals in other taxa. As a result, to detect chemoattraction in non-model species, very different methodologies and tools have been used, some of which are based on the estimate of sperm accumulation towards a chemoattractant rather than a proper chemoattraction test, in the sense of [16]. Despite not being definitive in unravelling the underlying mechanism (as sperm accumulation can be the result of such alternative mechanisms as sperm trapping or sperm chemokinesis alteration, see Discussion), measuring sperm accumulation is a valuable approach to understanding the role and the evolutionary consequences of FRF in the fertilization process. A measure of sperm accumulation induced by the FRF is indeed usually simple to obtain, and it has a clear endpoint (cell count). Additionally, independent of the mechanism(s) leading to sperm accumulation, the result of it (more sperm found where FRF is present) is relevant for understanding whether FRF is involved in helping the sperm to find and reach the fertilization site, thus affecting the chances of fertilizing the eggs). Using amphibian sperm, Burnett and colleagues [21] neatly showed how sperm accumulation and sperm movement towards FRF are different types of measures and are tested using different devices. Clearly, the use of one or the other approach (testing sperm accumulation or sperm chemokinesis) responds to different questions and therefore needs to be carefully evaluated by the researchers based on their research aim. As the majority of tests done in non-model taxa need to respond to the question of whether fertilization is facilitated by the presence of FRF, the first approach to use is the one that quantifies how many sperm reach the FRF (or the eggs surrounded by the FRF) rather than how this result is achieved. Therefore, sperm accumulation assays have been extensively used with various chemoattractants, and more recently with FRF, as they are relatively rapid and allow easy interpretations of the results. Some examples of these tools used in non-model species are capillaries, PVC tubes [22], or Transwell^®^ cell migration assays [4]. While at the species-level these approaches are effective for their scope, they are so diverse that they cannot provide a standard baseline approach adaptable to species specific needs, thus making the comparison among results difficult.

Here, we present a simple, reusable, and inexpensive tool designed to detect sperm accumulation under different conditions, suitable to be used with sperm from different species with only minor modifications. We developed and tested a ‘sperm selection chamber’ that circumvents most of the methodological problems faced when studying sperm chemoattraction through sperm accumulation. Specifically, when using FRF as a chemoattractant, there is often the additional problem of having a limited quantity of available FRF, and this is accentuated in small animals, including small vertebrates and insects [1]. We developed a device that can solve these problems by using small volumes. Our sperm selection chamber consists of a 3D printed multi-well chamber where a central well is connected to three smaller wells by three channels. One of these wells is used to add sperm in the chamber, another to add the chemoattractant (FRF) to create a chemical gradient to attract the sperm, and the third well and its channel are used as a control. The device thus allows in one single trial the selection, collection, and use (e.g., for further testing or for in vitro fertilization) of sperm that reach the chemoattractant well or the control solution’s well, therefore offering an internal control.

We tested the sperm selection chamber using the zebrafish, *Danio rerio*. In this species, a previous study has demonstrated an effect of FRF on sperm traits (velocity, trajectory, and longevity) in the absence of a gradient. Specifically, in the presence of FRF, sperm exhibit increased velocity, motility, and longevity but decreased linearity. This effect is dependent on the specific combination of males and females used (i.e., the same fluid has a different effect on sperm from different males and vice versa), indicating post-copulatory sexual selection processes mediated by the FRF [23]. However, sperm chemoattraction by FRF has never been demonstrated in this species. Here, we tested both the effectiveness of the sperm selection chamber and the presence of sperm accumulation towards FRF in the zebrafish. We tested a range of experimental conditions to identify a protocol that maximizes the efficiency of the sperm selection chamber for this species. Finally, to test whether the putative chemoattractive response is correlated with the intrinsic sperm quality, we tested whether the characteristics of the sperm (number and quality) affect the number of sperm reaching the FRF.

## 2. Materials and Methods

### 2.1. Subjects and Fish Maintenance

We used wildtype zebrafish from the Tübingen line, bred and maintained in the zebrafish facility of the Biology Department at the University of Padova. Fish were maintained under standardized laboratory conditions (12:12 light/dark cycle, 28 ± 1 °C water temperature) and fed three times a day with commercial flake food and live *Artemia salina* nauplii. All fish used were adults of 8–10 months of age.

### 2.2. Design of the Sperm Choice-Chamber

**Figure 1:**
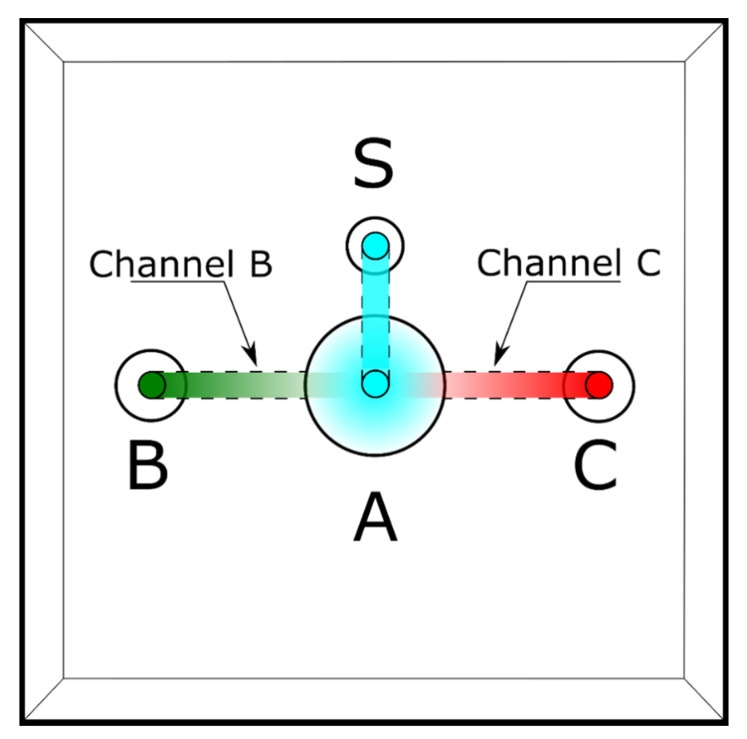
A schematic representation of the sperm selection chamber used. A: the main chamber where sperm are activated. S: The well where the sperm are added. B and C: the wells used for the chemoattractant (FRF) and control solution (water). More details, a step-by-step protocol, and the 3D project are available in Supplementary Materials.

We first designed a device (hereafter ‘sperm choice chamber’) with the shape and dimensions appropriate to handle the ejaculate and the FRF produced by *D. rerio*. During the design process, we aimed to meet the following: (1) the possibility to use small volumes of FRF (2–5 µl) as the chemoattractant agent, (2) the possibility to differentiate sperm sub-populations within those that respond to the FRF, and (3) the possibility to collect sperm once they reach the chemoattractant well. This device (Figure 1) has a central well that branches into 3 different channels, each with a well at the end. Water (activating solution) is in the central well (A), and the chemoattractant to one of the other two wells (B or C). The chemoattractant creates a gradient between this well and the central well (channel B), while no gradient is present in the other, control, channel (channel C). We estimated that 90 s are enough for the gradient to form after visually checking the time a dye needed to diffuse in the central well (see Supplementary Materials and Supplementary Video 1). Then, sperm are added to the third well (S) and, from there, reach the central well, where they will be activated by the water. Sperm that respond to the chemoattractant and reach the chemoattractant well (B), or that randomly reach the control well (C), can be recovered using a pipette. Further details on the chamber, such as the size and depth of the wells and channels, are available in the Supplementary Materials (Figure S1), along with a 3D file that can be used to directly print the device or can be modified to meet specific needs (e.g., modifying the length of the channels). The 3D project (Supplementary Material: file 2WellsC.stl) was created with the software Autodesk^®^ Fusion360 (San Rafael, CA, USA). The physical copy of the choice chamber was 3D printed in biological-inert resin (VisiJet^®^ Crystal, EX 200 Plastic Material, USP Class VI certified for medical applications. 3D Systems, Rock Hill, South Carolina, USA) with lost-wax technique (high definition 3D printing service, 29-micron accuracy declared). Because our device was developed for testing the sperm chemoattractant response to the FRF in the zebrafish, in which sperm are activated in water, we will refer to FRF as the chemoattractant and water as the main solution filling the device and acting as a control solution. However, these solutions can be substituted with other solutions in which sperm can swim, and the FRF and control solution can be replaced with other chemoattractant or any solution of interest.

### 2.3. FRF Collection

Female reproductive fluid was collected from 16 adult females using a standardized protocol, modified from [23]. Each female was isolated from males the day before the FRF extraction in 1 L tanks but with visual and olfactory access to a male. The morning after, each female was anaesthetized in MS222 (tricaine methanesulfonate, Sigma-Aldrich, Burlington, MA, USA; 0.17 g/L) and placed on a flat surface. The abdomen of the female was gently pressed to release eggs along with the FRF. We added a fixed amount of water to the eggs (1 µL/egg) and immediately collected the solution with a micropipette. We did not collect FRF in cases of contamination with feces or urine, or when broken eggs were present. The FRF solution was centrifuged at 16,100× g for 2 min, and the pellet was discarded to remove debris. FRF was then placed in a test tube and stored at −80 °C until used [24].

### 2.4. Sperm Collection from Males

To obtain ejaculates we followed the protocol described in Poli et al. [23]. Briefly, the day before each experiment, males were isolated in single-sex tanks. On the day of the experiment, each male was anaesthetized in MS222 and placed on a wet sponge under a dissecting microscope. The genital area and the abdomen of the male were dried to avoid accidental activation of sperm by water. The ejaculate was then collected in a glass capillary by gently pressing the abdomen of males. The ejaculate was diluted in 80 µL of Hank’s solution [25]. We then pooled together the ejaculates from three different males after adjusting their concentrations (based on CASA results) so that the sperm pool contained an equal proportion of sperm from the three males used. Sperm pool concentration was assessed with a LUNA-FL™ Cell Counter (Logos Biosystems, Gyeonggi-do, Korea). We obtained a total of 16 different sperm pools, using a total of 46 males (two males were used twice in two different pools). Using a pool of ejaculates obtained from three different males was important to minimize the confounding effects of the specific effect of FRF–sperm interactions due to female × male combinations in this species [23] as well as in others [1].

### 2.5. Test of the Sperm Choice-Chamber

We tested our sperm choice chamber at different combinations of time and volume, that were: (i) the time between sperm activation—when the sperm are loaded into the device—and sperm recovery (hereafter, time from activation: TA); and (ii) the volume of solution recovered, which allows selecting for sperm that moved more or less deeply inside the channels (hereafter, sampling volume: SV). TA and SV affect how many sperm are collected and what sub-population of sperm, based on its response to the FRF gradient. Smaller SV will allow collecting only sperm closer to the chemoattractant well (i.e., sperm that responded well to the chemoattractant), while larger SV will also allow collecting sperm closer to the central well (i.e., sperm that responded less to the chemoattractant). Similarly, with a shorter TA, only sperm that responded quickly to the chemoattractant or that swam faster within the channel will be collected, while with a longer TA, slower and less responsive sperm will be also collected. One of our aims was to determine under what conditions of SV and TA the chemoattractant effect of FRF was maximized compared with the control solution (i.e., when the difference between the number of sperm collected from the FRF’s well and sperm collected from the control’s well was the greatest).

To use the sperm choice chamber, we developed a simple protocol consisting of a few steps (see Supplementary Figure S3 for a visual diagram of the experimental protocol and Supplementary Video 1), as described below:

1. Add water through the three smaller wells (S, B, and C; 12 µL each) using a micropipette (P10 or P20). This step fills chamber A and avoids air bubble formation in the channels;
2. Four microliters of each of the chosen solutions (FRF as chemoattractant and water as control) is added to wells B and C. At this point, the two solutions will start to gradually mix with the water already present in the device. The FRF gradient will gradually form over 90 s, starting from the well, through its channel (channel B or C), to the central well (A);
3. Twenty microliters of sperm pool solution is gently added to the sperm well S;
4. After the desired amount of time (TA), sperm can be collected at the desire sampling volume (SV) through the two collection wells (B and C) using a micropipette (P10 or P20);
5. The number of sperm collected are counted to assess sperm accumulation.

A total of 16 trials were performed. For each trial, we used a different pool of sperm and a different FRF, therefore always using unique combinations of sperm pool × FRF. In each trial we tested nine different combinations of TA (20, 30, and 40 s) and SV (2, 2.5, and 3 µL). The order in which each combination of TA and SV was tested was randomized across trials. Similarly, which well was used for FRF or water (left or right, B or C in Figure 1) was randomized across trials. Trials were performed blind to the identity of the solution used. The sperm choice chamber and the water used to fill the main chamber were maintained at 28 °C (the temperature at which fish are maintained). The sperm choice chamber was carefully washed with distilled water (using a disposable Pasteur plastic pipette) and dried with compressed air between trials. To measure the number of sperm recovered from each well (FRF and water), one can choose a preferred method, including counting with CASA software, cell counters, or manually with a hemocytometer. To speed up operation we used a LUNA-FL™ cell counter (Logos Biosystems, Gyeonggi-do, Korea) following the manufacturer’s instruction [26]. Each measure was repeated three times (*R* = 0.91; S.E. = 0.009, *p* < 0.001) on the same slide, and the average was used.

### 2.6. Repeatability Test

To validate the reliability of the sperm choice chamber as a research tool, we tested the repeatability (ICC, intraclass correlation [27]) of the data obtained using the sperm choice chamber with the protocol we developed. The repeatability of the sperm choice chamber was estimated in a total of 10 trials (i.e., 10 different combinations of sperm pool and FRF), replicated twice, using a TA of 20 s and a sampling volume of 3 µL. To this end, two different ejaculate pools were tested as above, with five different FRF each. Repeatability assays were done blind.

### 2.7. Ejaculate Quality Assay

Sperm quality was assessed using a sperm tracker (CASA, Hamilton-Thorn, Beverly, MA, USA, CEROS version 12.3, 60 fps, recording time = 0.5 s). Sperm were activated with water (1:2 ejaculate solution to water dilution), immediately placed on a Leja^®^ slide, and (focusing on the center of the slide) the following parameters of sperm motility were recorded: sperm curvilinear velocity (VCL), sperm linearity (LIN), beat-cross frequency (BCF), and motility (MOT, percentage of motile sperm). Sperm longevity was also assessed as the time elapsed from activation until ≤20% of cells in the field of view were motile [23]. Each sperm sample was measured twice, and the average of the two measures was calculated. Then, the average of the three ejaculates within each sperm pool was calculated for each motility parameter. This average value was used to test the correlation between pool motility parameters and the number of sperm retrieved from the collection wells (see below).

### 2.8. Statistical Analysis

The repeatability (ICC) was estimated using the ‘rptR’ R package [28]. We used the number of sperm retrieved from the collection wells filled with FRF over the total number of cells collected in the two wells as the estimated parameter. The rptR package estimates repeatability from generalized linear mixed-effects models fitted by restricted maximum likelihood (REML).

We first assessed whether the number of sperm retrieved from the FRF well was different from the number of sperm retrieved from the control well. We fit a generalized linear mixed model (GLMM) with binomial distribution and logit link function in the ‘lme4’ package [29]. The combined number of sperm collected from the two wells (water + FRF) was the binomial total, and the number of sperm in the FRF well was the response term. In the model, the response variable was modeled as number of successes and number of failures (number of sperm in the FRF well as success and number of sperm in the control well as failures, see attached R code description in Supplementary Materials). Model parameters were estimated with the Laplace approximation of the log-likelihood. The model included a fixed intercept and the sperm pool ID as a random effect. An observation-level random effect with a separate level for each measurement was used to correct for overdispersion (final dispersion = 0.97). Residuals distribution was visually checked to meet the assumptions of the model.

When testing the effect of TA, SV, and sperm pool concentration, we fit a GLMM as above. The model included a fixed intercept and sperm pool ID as a random effect. SV, TA, and their interactions were also entered as categorical fixed factors. Sperm pool concentration was entered in the model as a covariate. As the model was overdispersed, we added an observation-level random effect with a separate level for each measurement (final dispersion = 0.97).

**Figure 2:**
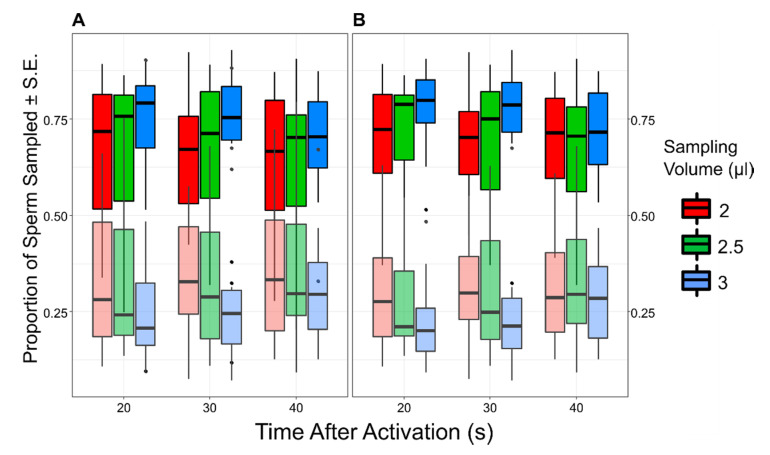
The boxplots show the proportion of sperm collected from the FRF (bright colors) and water (dull colors). Each box represents the value for a specific combination of time from activation (TA) and sampling volume (SV). In (**A**), the complete dataset is shown. In (**B**), data from trial 3 and 13 have been removed.

To estimate whether there was a correlation between ejaculate quality (CASA parameters and longevity) and sperm accumulation in the FRF, we considered only one combination of TA and SV (i.e., 20 s and 3 µL), in which the proportion of sperm retrieved from the FRF’s well over the total of sperm sampled was higher (see Figure 2). We then calculated the Pearson correlation between the total number of sperm collected from the FRF’s well (square root transformed) and the sperm quality parameters estimated for each pool.

Means are reported with their standard error (S.E.).

All analyses were performed with R (version 4.0.3), and the code used is provided in Supplementary Materials.

## 3. Results

The repeatability of the method (see also Figure S4) was significant (*R* = 0.63; S.E. = 0.26, *p* = 0.016). After removing a single measure where the number of sperm in the control well was higher than the number of sperm in the FRF well (Trial no. 5), the repeatability increased (*R* = 0.88; S.E. = 0.12, *p* < 0.01).

**Figure 3:**
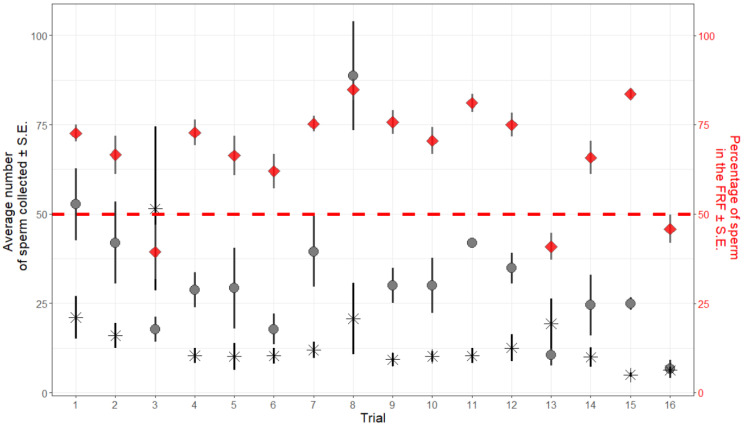
The average (± S.E.) number of sperm collected in the nine replicates of each trial. The number of sperm collected in the FRF (circles) is higher than in the control (stars) in all but two cases (trials #3 and #13). Red diamonds represent the average percentage of sperm collected in the FRF in the nine combinations of TA and SV (± S.E.) within each trial. The red dashed line represents 50%: values (diamonds) above the line represent trials where the majority of sperm (>50%) that reached the wells were collected from the FRF well.

In 122 cases out of 144 (16 trials each repeated 9 times), the number of sperm collected in the FRF was higher than that of sperm collected in the water. The opposite, i.e., more sperm were retrieved from the control well, was seldomly observed and was confined to 2 trials out of 16 (see Figure 3). We interpreted this effect as a probable operator’s error in de-coding the sample identity (the experiment was performed blind) and present results excluding these trials. Including or excluding these two trials did not substantially change the general result about how sperm respond towards FRF. However, when including these two trials, the effect TA becomes non-significant (TA: *p* = 0.28), despite showing the same trend. Results obtained with the full dataset are reported in the Supplementary Materials.

Sperm retrieved from the FRF’s well were significantly more than those recovered from the control’s well (FRF = 35.10 ± 2.46; water = 11.69 ± 1.06; GLMM: intercept *z* = 9.01, *p* < 0.001). When sperm pool concentration, SV, and TA were included in the model, sperm retrieved from the FRF’s well were more than the control (intercept *z* = 3.02, *p* = 0.003), and TA (χ_22_ = 6.29, *p* = 0.043) had a significant effect on the relative number of sperm collected from the two wells, with relatively more sperm found in the FRF’s well at shorter TA (Figure 2). SV (χ_22_ = 5.52, *p* = 0.063), the interaction between TA and SV (TA x SV, χ_42_ = 2.47, *p* = 0.651), and the initial number of sperm introduced in the device (sperm pool concentration, χ_12_ = 0.49, *p* = 0.486) did not have a significant effect.

We analyzed whether the intrinsic quality of sperm (motility and longevity) affected the outcome of putative sperm chemoattraction. We found no evidence of a correlation between any of the sperm quality parameters we considered and the absolute number of sperm collected from the FRF well (VCL: *r* = 0.37 *p* = 0.19; LIN: *r* = −0.14 *p* = 0.62; BCF: *r* = −0.29 *p* = 0.31; MOT: *r* = −0.03 *p* = 0.91; Longevity: *r* = 0.075 *p* = 0.80).

## 4. Discussion

We showed that sperm accumulate towards the female reproductive fluid in the zebrafish. Using the sperm selection chamber that we developed, we found that, on average, sperm were 3 times more numerous in the FRF compared with those in the control. The time from activation, TA (i.e., the time between when sperm were added and retrieved), affected how many sperm were recovered in the FRF compared with the control solution; sperm were relatively more abundant in the FRF when collected after a shorter TA, and even if non-significant, there was a trend for finding relatively more sperm in the FRF with larger sampling volumes (SV). These results allowed us to develop an ad hoc repeatable protocol by combining specific TA and SV to maximize the efficiency of our sperm selection chamber. Interestingly, the concentration of the sperm pool added to the device did not have any effect on the relative number of sperm collected. Similarly, none of the estimates of sperm quality were correlated with the number of sperm collected in the FRF.

It has to be noted that our experiment was designed to assess sperm accumulation in the presence of FRF as chemoattractant, and as such it cannot distinguish across different mechanisms at the base of the sperm accumulation—namely, sperm chemotaxis, chemokinesis, and sperm trapping. However, we may speculate that the sperm accumulation in our experimental design is most likely due to sperm chemotaxis rather than sperm chemokinesis or trapping. Indeed, chemokines is accounted for by the design itself of the chamber as it provides a ‘choice’ for the sperm between the chemoattractant and the control (see [16]). Second, sperm trapping occurs when sperm cannot swim back once they reach a certain place and can be due to different causes, including sperm hyperactivation, or decreased sperm motility [16,30]. Hyperactivation can be excluded as it is not described in zebrafish sperm (or any other fish species), and we have never observed it in our experiment, nor was it observed in [23] (without a gradient). Interestingly, Poli et al. [23] reported that in the presence of FRF sperm swim faster but in a more circular trajectory (decreased linearity), suggesting that FRF can affect sperm swimming direction. On the other hand, this change in sperm behavior could also favor, at least to some extent, sperm being ‘trapped’ in the FRF well once they reached it. In this study, however, the number of sperm found in the FRF did not significantly increase with time, something that one would predict if the sperm accumulation in FRF well was caused by sperm trapping mediated by this change in sperm swimming linearity. Therefore, together all these considerations make it more plausible that the sperm accumulation we observed in this study was due to sperm chemotaxis, but further investigation is needed to distinguish among different mechanisms, as suggested in [16,31], and for this reason we cautiously use the terminology ‘putative evidence of chemotaxis’ to refer to our results.

Most of the knowledge about sperm chemoattraction comes from mammals and invertebrate broadcast spawners, for which we now know in detail the molecular mechanisms involved, the resulting changes in swimming parameters, the hydrodynamic role of the fluidic environment, and its fitness consequences [10,16,31,32,33,34,35,36,37,38,39,40,41,42]. Most of the tools aimed at testing sperm chemoattraction have been developed for species (e.g., [17,20,31,43,44,45,46]) in which sperm are motile for long periods and ejaculate is generally abundant.

In fishes, the effects of FRF on sperm performance (such as sperm motility, swimming velocity, longevity, and trajectory) is known to be very common (reviewed in [47]), but direct evidence of chemoattraction of sperm towards FRF is still limited (reviewed in [4,47,48,49]). Our findings add to this gap by suggesting that zebrafish sperm are attracted to the eggs through the female reproductive fluid, indicating, as suggested by previous studies [4,50], that FRF per se (without eggs) exerts a putative chemoattraction on sperm. As recently pointed out by Kholodnyy and colleagues [49], one possible reason for the general lack of knowledge in sperm chemoattraction in fish (but it applies also to other taxa) might be sought in the experimental challenges faced by researchers. Here, we provide a solution to this problem, demonstrating that our inexpensive and easy-to-use ‘sperm selection chamber’, which offers reliable and repeatable results, can simplify and standardize future experiments on sperm chemoattraction behavior in fish and other less-studied systems, such as non-mammal species, including invertebrates (e.g., insects). We successfully used the sperm selection chamber in a model species, the zebrafish, in which both of the most common limitations in this type of study are present: small volume of ejaculate and short sperm longevity.

In our trials, we observed that the proportion of sperm in the FRF compared with control was maximum when the time between activation and collection (TA) was shorter (20 s), and it decreased as TA increased (30 and 40 s, see Figure 2). This suggests that while the absolute number of sperm retrieved in the FRF did not change over time (from 20 to 40 s; GLMM, *F*_2,110_ = 0.95, *p* = 0.388), those in the control increased as TA increased (GLMM, *F*_2,110_ = 4.78, *p* = 0.010), reducing the differences between FRF and control. This result may be due to sperm having the chance to randomly accumulate in the control channel with time, while those responding to FRF are doing so quickly at the onset of motility (within 20 s) and allowing more time does not translate into more sperm responding to the FRF. Indeed, sperm responding to a chemotaxis gradient may be subjected to receptors saturation, and hence they may reduce their response movements with time, as shown in sea urchins [16,51] and in mammals [52]. Alternatively, the FRF gradient may not be constant over time, resulting in the intensity of the attractant weakening with time. This might lead to sperm migrating more randomly to (and thus accumulating in) both wells, progressively reducing the bias towards the FRF well. This hypothesis is unlikely in our experiment as the difference in time between the two extreme time measures (40 s and 20 s) was very short. However, we cannot rule it out, and only a formal estimate of gradient formation and stability over time would clarify this. We also found (albeit non-significant) relatively more sperm when collecting a larger sampling volume (SV). Different sampling volumes reflected different sampling regions in the two channels; with 2 µL, only sperm that swam deeply inside the channel towards the well (B, Figure 1) were collected, whereas with 3 µL also sperm close to the channel entrance (i.e., closer to the central well A, Figure 1) were collected. That means that by changing the SV in our device, one can select sub-populations of sperm, potentially differing in their ability, or velocity, to respond to the chemoattractant. This may become handy for future research that wants to study mechanisms and consequences of intra-ejaculate variation in this context.

Surprisingly, neither the sperm pool concentration nor any of the sperm quality estimates (sperm swimming speed, swimming linearity, sperm beat frequency, motility, and longevity) were correlated with how many sperm were collected in the FRF well. Together these results may suggest that, under our experimental conditions, only a portion of sperm can respond to the FRF and that this portion is independent of the initial number of sperm or their quality. This is corroborated by findings in other taxa, as, for example, in humans where only a tiny fraction (2–12%) of sperm is chemotactically responsive [53]. We used pools of sperm of different males to avoid effects due to male × female effect (as observed in [23]), but by doing this we have also potentially reduced the variance among pools in sperm quality traits, thus potentially reducing our power to detect any effect of ejaculate quality in the response to FRF. Using sperm pools allowed us to standardize the experimental conditions among trials, which is necessary when testing a new tool and developing a protocol of use; nevertheless, future work should aim to confirm whether this lack of association between sperm quality and response to FRF is present when single ejaculate × FRF is used.

The simple experimental procedure we present requires a small volume of FRF and allows sperm successfully attracted by FRF to be collected after only 20 s. After this time, sperm in the zebrafish are still viable and motile [23], which was also observed in this experiment and can thus be used for further measurements or in subsequent experimental procedures (e.g., in vitro fertilization). For example, sperm attracted by FRF may have a lower proportion of DNA fragmented, which has been linked to an increase in embryo viability in the zebrafish [54] and in the rainbow trout [55]; thus, considering other sperm characteristics, researchers can effectively test whether FRF is able to select certain phenotypes of sperm, a possibility that we are currently investigating. We think that the experimental tool and the ad hoc protocol we developed have the potential to increase the variety of research linked to the reproductive biology of *D. rerio*. We believe that our sperm selection chamber can also help researchers in studying the interaction effect between sperm and FRF through chemoattraction, expanding the array of conditions and non-model species in which this interaction can be tested. The sperm selection chamber is small enough to be used with a small amount of FRF and ejaculate and with sperm that swim for a short time and within a short distance, and it allows sperm that responded to the chemoattractant agent to be collected. Moreover, modifications of the protocol may fill our methodological gap and further disentangle the effect of chemotaxis, chemokinesis and trapping—for example, implementing a descending chemoattractant gradient assay (where sperm are suspended in a chemoattractant solution and their accumulation at the bottom of a chemoattractant gradient is compared with their accumulation where the chemoattractant concentration is constant [16]). Other devices have also been used to assess sperm chemoattraction. We would like to compare our device with the most similar one (for the type of assay and ease-of-use), the Transwell^®^ (used in [4,9]). Modified cell migration assay using Transwell^®^, consists of an outer well in which an inner well with a membrane is inserted. The membrane can have different diameters of the porous material that allows sperm through it into the outer well. FRF is added in the outer well and water (or appropriate solution) in the inner well, and sperm are then added in the inner well. After a certain period of time, the inner well with the membrane is removed, and sperm that have been attracted by the FRF can be collected in the outer well. This assay has been used so far in the context of FRF in two fish species ([4,9]). The first difference is in the volume of solution required by the Transwell^®^ (100 µL or 200 µL need to be added to the outer well containing FRF), which cannot then be used when the FRF volume retrieved from the female is small (e.g., in the zebrafish or other small animals, such as insects, FRF collected is in the order of a few µL). Second, the Transwell^®^ does not allow an internal control as is allowed in the tool we developed. Indeed, our device offers the possibility of testing a simultaneous response to different chemoattractants. Third, our device can be easily modified by adjusting the 3D project—for example, making the wells bigger or the channel longer based on available data on sperm movement in the desired species (see also Supplementary Materials). In addition, the small size makes it easy to be heated or cooled with simple tools, such as placing the chamber on a heated pad with which temperature can be controlled, or the device can be transported easily in the field for on-the-spot assays, as it does not require a microscope, and experiments can be performed with only a table and a pipette (sperm can be collected from the wells and stored appropriately in tubes to be counted with the preferred method at a subsequent time).

Among the many possibilities, this device can help to (i) evaluate the presence of sperm accumulation towards FRF or other chemicals, (ii) evaluate female × male interactions in gamete attraction, (iii) distinguish and collect sperm selected vs. non-selected or selected by different chemoattractants, and (iv) test the disruptive effect of contaminants and pollution in gamete chemotaxis. Moreover, both the protocol and the sperm selection chamber can be easily modified to account for other species-specific needs—for example, increasing the length of the channels, the overall capacity of the chamber, or the number of wells (see Supplementary Material: file 4WellsC.stl). In conclusion, we believe our device will lay the foundation for future research aimed at understanding the role of FRF and, more generally, sperm chemoattraction during the fertilization process.

## Data Availability

The dataset is available as Supplementary Materials.

## Supplementary Materials

The following are available online at https://www.mdpi.com/article/10.3390/cells10092472/s1, Supplementary Information Devigili at al.docx: a text file with the following information: Figure S1: Detailed project of the sperm selection chamber, Figure S2: Instruments used for the experiment, Figure S3: Working protocol details, Figure S4: A graphic representation of the repeatability test. 2WellsC.stl and 4WellsC.stl, selection chambers 3D printable files. Video S1: The video shows how a gradient is formed within the central chamber. Dataset.xlsx: original dataset. Analysis and Figures Code.R: the R code used for statistical analysis and figures generation. R Markdown Code.html: a detailed description of the R code used.
