# Supplementary material for: Sperm Accumulation Induced by the Female Reproductive Fluid: Putative Evidence of Chemoattraction Using a New Tool
Source: Cells. 2021 Sep 18;10(9):2472. doi: 10.3390/cells10092472 (PMC8467055; doi:10.3390/cells10092472)

Supplementary Material

Sperm accumulation induced by the female reproductive fluid: finding putative evidence of chemoattraction with a new tool

Alessandro Devigili[1*], Silvia Cattelan[1], Clelia Gasparini[1]

[1]Department of Biology, University of Padova, via Ugo Bassi 58/B, Padova, Italy.
*Correspondence: alessandro.devigili@unipd.it

Figure S1. Detailed project of the sperm selection chamber. All quotes are in millimeters. Initially the **main well** is filled with water (or the appropriate solution). The **sperm well** is used to add the ejaculate to the chamber, that will thus reach the central, main, well where they will be activated. The **FRF** (chemoattractant) well and **water** (control) well are first used to add the chemoattractant and the control solution and later to collect the sperm. Note that the sperm selection chamber 3D project has wells labelled with different letters to facilitate keeping track of loading and collecting operations.

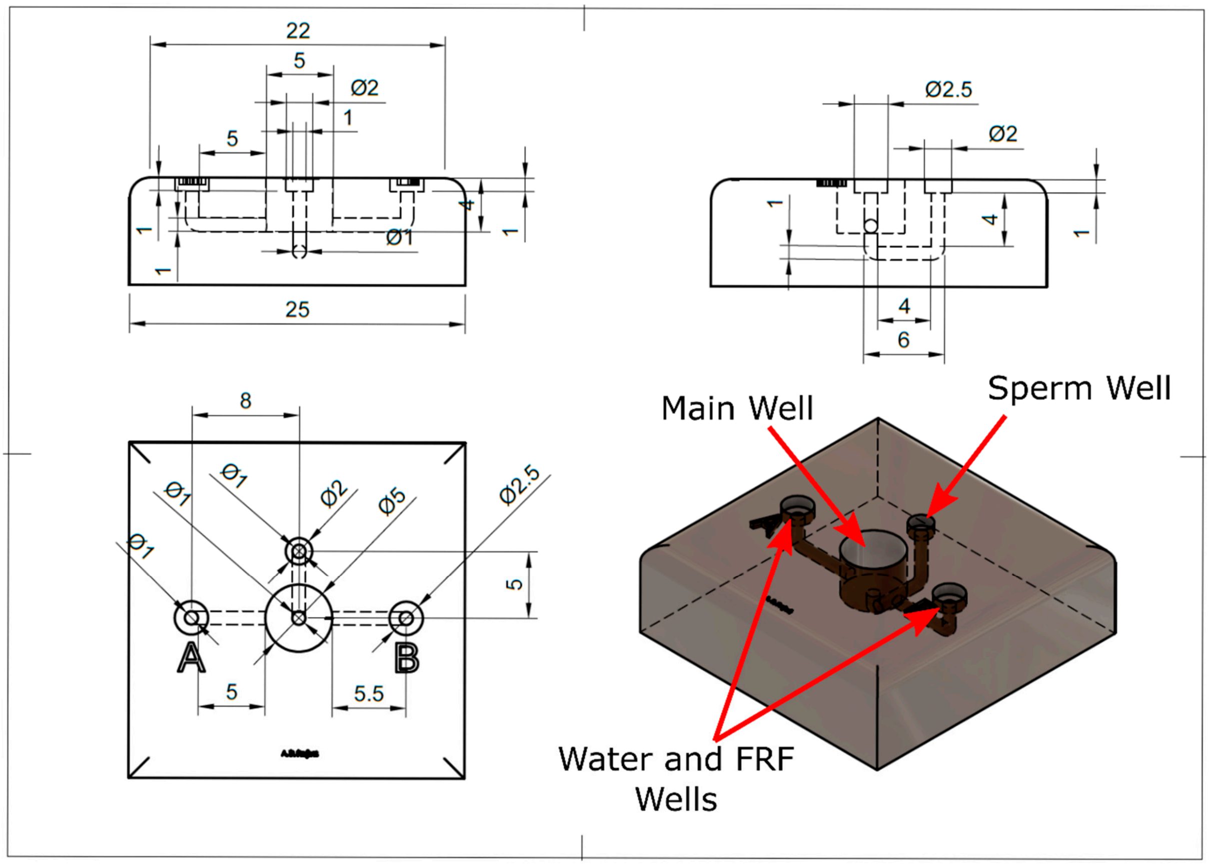

Figure S2. A photograph of the instruments used for the experiment. The sperm choice chamber was used on a heated platform which kept the temperature at 28° C. P20 and P10 micropipettes were used with 2-200 µl micropipettes tips. Sperm were counted on a reusable counting slide for the Luna system (Logos).

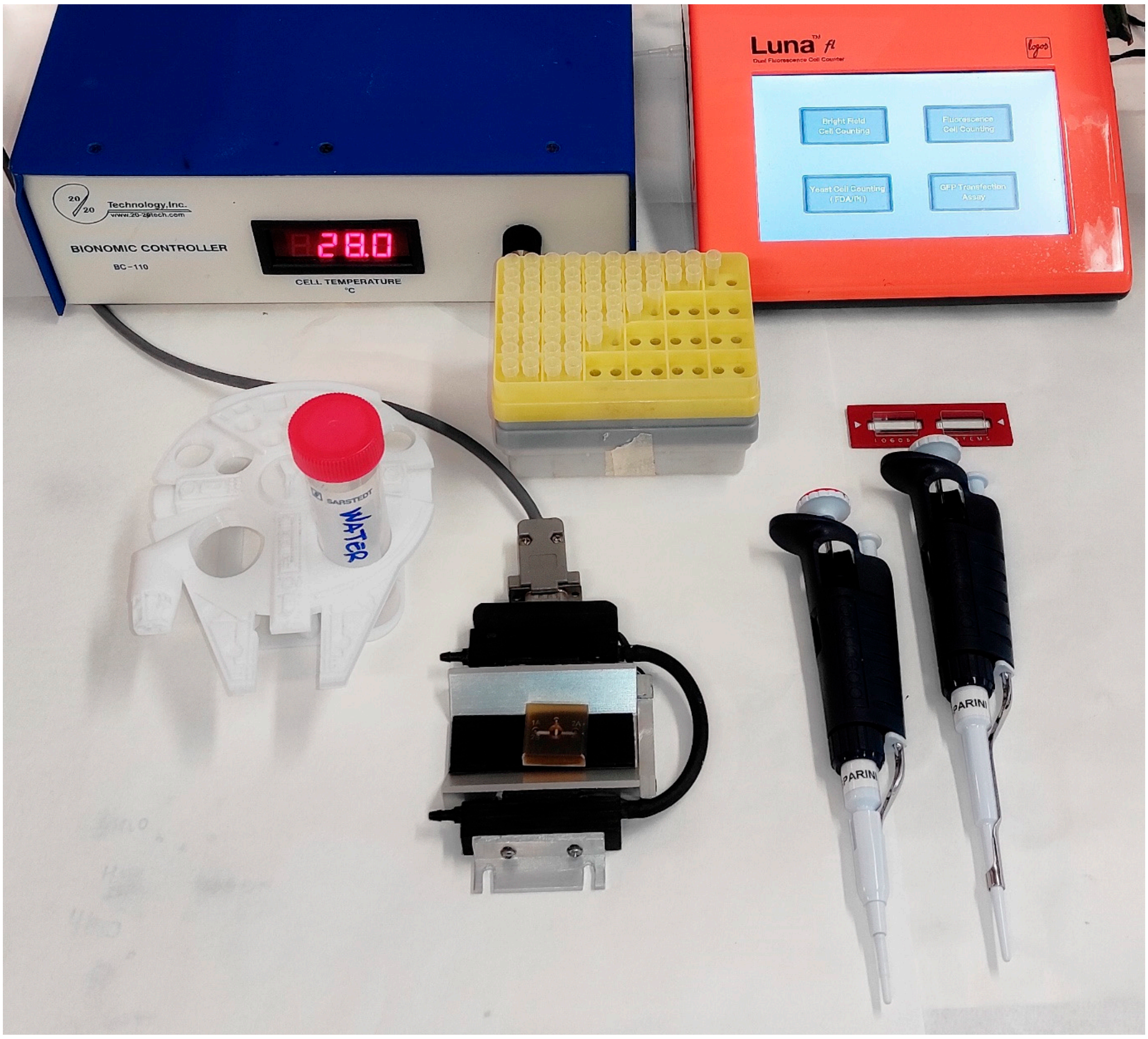

Protocol details

Figure S3 visually represents the steps of the working protocol (see also main text).

1. The sperm choice chamber can be previously heated or cooled placing it on a temperature controller for microscope slides (as in figure S2) or on a temperature controlled plate.
2. The central chamber (A) has to be previously filled (with an activating solution, e.g. water) through the wells S, B and C (12 µl each) to ensure no air bublles form in the channels.
3. Testing solutions (e.g. female reproductive fluid and water) are then added (4 µl) through the wells B and C in to the channels.
4. After the gradient is formed (about 90 seconds), sperm are added through the well S (20 µl).
5. After the desired amount of time (TA), sperm can be recovered from wells B and C.

We suggest to use P10 and P20 (with P20 tips) micropipettes to perform these operations. The pipette's tip has to be hold firmly and inserted perpendicularly in the wells to ensure proper adhesion.

Preliminary, we evaluated the time needed to the chemical gradient to form. To this end, we visually estimated how long a loading dye (GoTaq® Green Buffers for PCR, Promega) needs to diffuse (see supplementary video 1). In our preliminary experiment 90 seconds were enough for the dye to reach the centre of the main well.

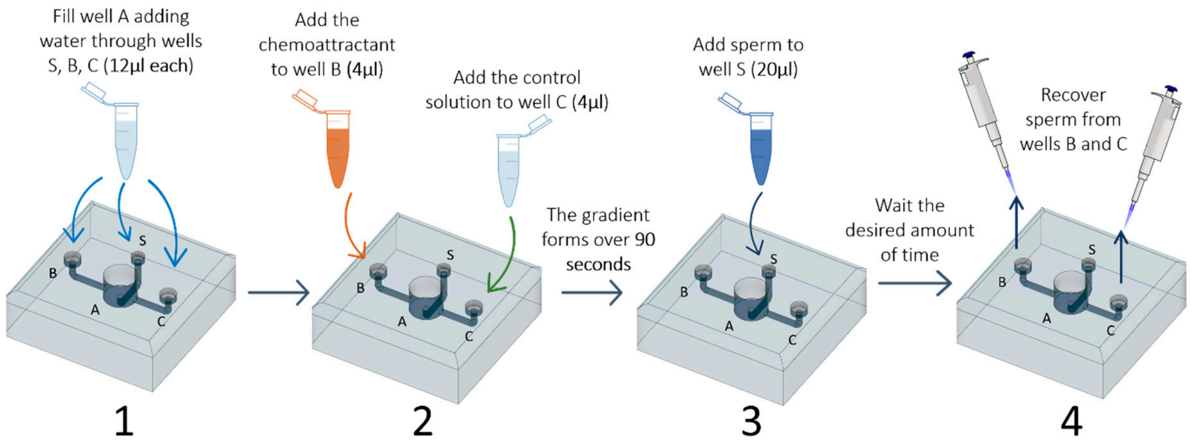

Supplementary results

When analyzing the full dataset (including the two trials where a de-coding error was probably made, see main text) the main result did not change. Sperm retrieved from the FRF's well were significantly more than those recovered from the control's well (FRF, N=32.46±2.39; water, N=14.66±2.01; intercept z=4.97, p<0.001). However, differently from the models that excluded these two trials (see main text), TA did not have a significant effect (SV: χ_2^2=2.04, p=0.360; TA: χ_2^2=2.55, p=0.279; SV*TA interaction: χ_2^2=0.41, p=0.520). Howeevr, the results are qualitatively similar, so that with higher SV and lower TA sperm collected in FRF are relatively more (see figure 3 in the main text).

Figure S4. A graphic representation of the repeatability of our protocol using the sperm choice chamber in standard conditions (TA= 20 seconds, SV = 3 μl). Each dot represents the percentage of sperm collected in the FRF channel in a single trial. Each trial was repeated twice. Different colours represent the two sperm pools used.

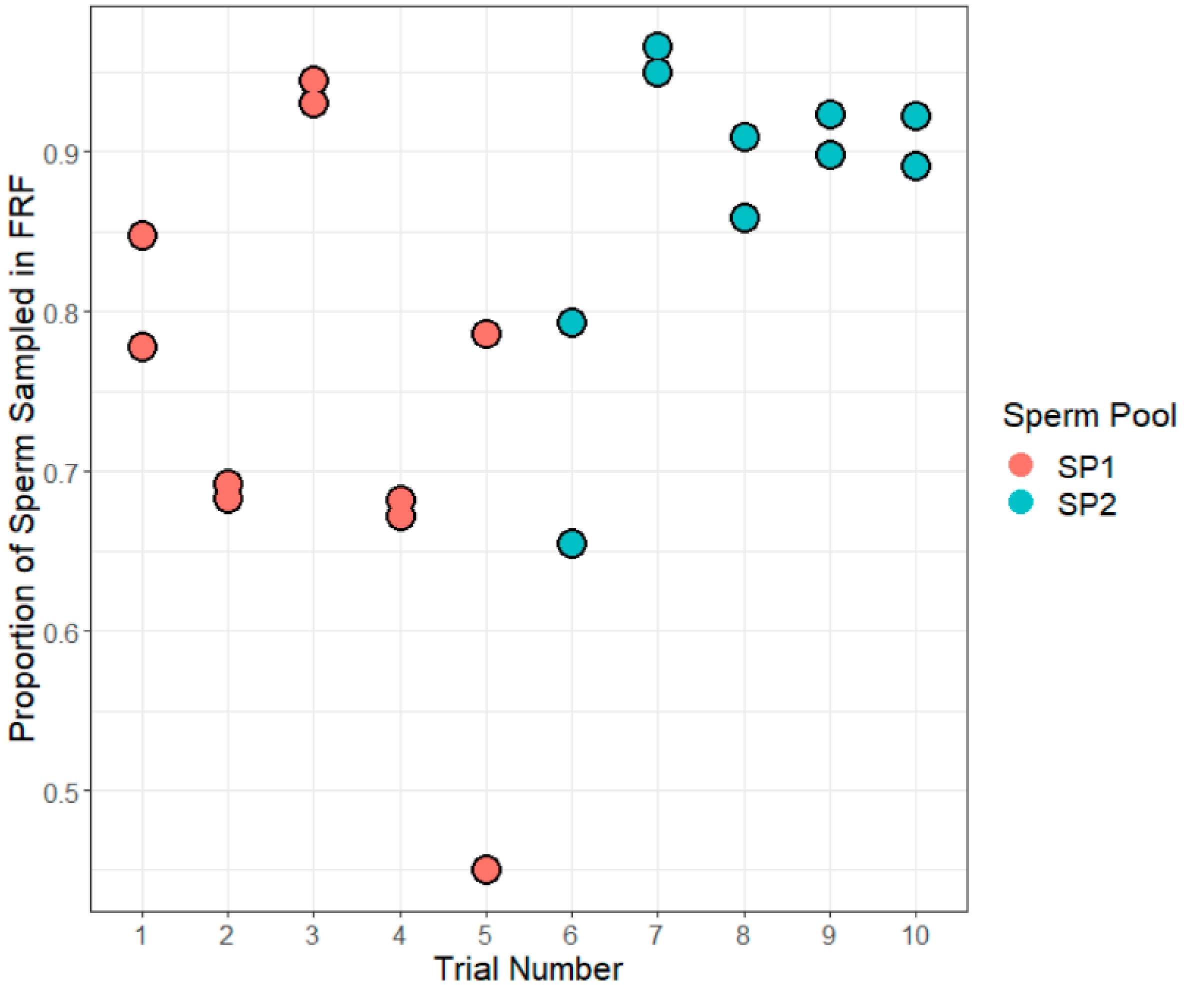

Supplement: Supplementary file 1 [file cells-10-02472-s001.zip › cells-1251641-supplementary materials.pdf]
