# Supplementary material for: Sperm Accumulation Induced by the Female Reproductive Fluid: Putative Evidence of Chemoattraction Using a New Tool
Source: Cells. 2021 Sep 18;10(9):2472. doi: 10.3390/cells10092472 (PMC8467055; doi:10.3390/cells10092472)
Supplement: Supplementary file 1 [file cells-10-02472-s001.zip › R Code Marckdown.html]

Sperm Accumulation Induced by the Female Reproductive Fluid: Putative Evidence of Chemoattraction Using a New Tool


# *Sperm Accumulation Induced by the Female Reproductive Fluid: Putative Evidence of Chemoattraction Using a New Tool*

####

## Authors:

### Alessandro Devigili1+, Silvia Cattelan1 and Clelia Gasparini1

1Department of Biology, University of Padova, via Ugo Bassi 58/B, Padova, Italy. +Correspondence: alessandro.devigili@unipd.it

## Below the code used in for analysing the data and making the figures

### Loading required packages

```
library(readxl)
library(lme4)
library(lmerTest)
library(dplyr)
library(tidyr)
library(ggplot2)
library(car)
library(effects)
library(rptR)
library(ggpubr)
```

### Loading the dataset

And get a description of the variables in the dataset

```
#setwd("") #set here your working folder
Variables<-read_xlsx("Dataset.xlsx",sheet=3) #see variables desscription
print(Variables, n=nrow(Variables))
```

```
## # A tibble: 23 x 2
##    `VARIABLE NAME` DESCRIPTION                                                  
##    <chr>           <chr>                                                        
##  1 Sperm_Pool      "The sperm pool used (unique)"                               
##  2 Trial           "Trials"                                                     
##  3 FRF             "FRF used (unique)"                                          
##  4 spermN          "Sperm pool average concentration"                           
##  5 VCL             "VCL measured. Average between the two measurements within m~
##  6 LIN             "Linearity"                                                  
##  7 BCF             "Beat Frequency"                                             
##  8 Longev          "Pool longevity (time to reach 20% motile sperm, calculated ~
##  9 MOT             "Motility"                                                   
## 10 First           "Wich side (1 or 2) was loaded and measured FIRST."          
## 11 Time            "Time from  Activation, \"TA\" (seconds)"                    
## 12 ul              "Sampling Volume \"SV\" (ul)"                                
## 13 AV_1            "Average count for side 1"                                   
## 14 AV_2            "Average count for side 2"                                   
## 15 Total           "The sum of AV_1 and AV_2"                                   
## 16 FRF_place       "Where was the FRF? In side 1 or side 2?"                    
## 17 Correction      "Scaling to apply to account for  sperm concentration correc~
## 18 C_1             "AV_1 corrected by concentration"                            
## 19 C_2             "AV_2 corrected by concentration"                            
## 20 Total_C         "The sum of sperm counted in the two sides (correced by conc~
## 21 FRF_n_C         "Average count in the FRF (corrected by concentration)"      
## 22 H2O_n_C         "Average count in the Water (corrected by concentration)"    
## 23 Perc_FRF        "Percentage of sperm in the FRF over all the sperm moved"
```

```
datachemotaxis<-read_xlsx("Dataset.xlsx",sheet=1) #get the data for analysis and figures
```

### Set the correct type of data

```
datachemotaxis$Trial<-as.factor(datachemotaxis$Trial)
datachemotaxis$First<-as.factor(datachemotaxis$First)
datachemotaxis$Time<-as.factor(datachemotaxis$Time)
datachemotaxis$ul<-as.factor(datachemotaxis$ul)
datachemotaxis$Sperm_Pool<-as.factor(datachemotaxis$Sperm_Pool)
datachemotaxis$spermM<-datachemotaxis$spermN/1000 #transform data to "number of thousands
datachemotaxis$ID <- seq.int(nrow(datachemotaxis))#add a variable with observation level number. This is useful when correcting for overdispersion
datachemotaxis<-datachemotaxis %>% mutate(Perc_H2O=1-Perc_FRF)#add a variable H2p Percentage 
datachemotaxis2<- datachemotaxis %>% filter(Trial!=3&Trial!=13) #remove trials with potential human errors

str(datachemotaxis) #to check the data structure
```

### Creating some useful function to check for overdispersion

These functions can be used to check the overdispersion of the models used. See link

```
##a function to later check for overdispersion of GLMM with binomial distribution
disp_glmer<- function(yourmodel)
{ n <- length(resid(yourmodel))
return( sqrt( sum(c(resid(yourmodel), yourmodel@u) ^2) / n ) )}

#another function to get overdispersion estimates with p from Ben Bolker FAQs
#https://bbolker.github.io/mixedmodels-misc/glmmFAQ.html#fitting-models-with-overdispersion
overdisp_fun <- function(model) {
        rdf <- df.residual(model)
        rp <- residuals(model,type="pearson")
        Pearson.chisq <- sum(rp^2)
        prat <- Pearson.chisq/rdf
        pval <- pchisq(Pearson.chisq, df=rdf, lower.tail=FALSE)
        c(chisq=Pearson.chisq,ratio=prat,rdf=rdf,p=pval)
}
```

## Do the RFR attract sperm? Or, are there more sperm in the FRF channel?

A significant intercept means more sperm in one channel compared to the other.

```
#create an object with successes (sperm in FRF channel) and failures (sperm in the water) to use as dependent variable in the model
Y<-as.matrix(round(select(datachemotaxis2,FRF_n_C,H2O_n_C)))
#Note that this new variable can be obtained in different ways.
#For example:
#Y<-(cbind(round(datachemotaxis2$FRF_n_C),round(datachemotaxis2$H2O_n_C)))
#Y<-as.matrix(round(datachemotaxis2[,c(21,22)]))

mod1<-glmer(Y~1+(1|ID)+(1|Trial),family="binomial",datachemotaxis2)
summary(mod1) #look at the intercept
```

```
## Generalized linear mixed model fit by maximum likelihood (Laplace
##   Approximation) [glmerMod]
##  Family: binomial  ( logit )
## Formula: Y ~ 1 + (1 | ID) + (1 | Trial)
##    Data: datachemotaxis2
## 
##      AIC      BIC   logLik deviance df.resid 
##    734.1    742.6   -364.0    728.1      123 
## 
## Scaled residuals: 
##      Min       1Q   Median       3Q      Max 
## -1.39743 -0.33905  0.01714  0.34320  1.53963 
## 
## Random effects:
##  Groups Name        Variance Std.Dev.
##  ID     (Intercept) 0.2312   0.4808  
##  Trial  (Intercept) 0.1418   0.3765  
## Number of obs: 126, groups:  ID, 126; Trial, 14
## 
## Fixed effects:
##             Estimate Std. Error z value Pr(>|z|)    
## (Intercept)   1.0498     0.1153   9.102   <2e-16 ***
## ---
## Signif. codes:  0 '***' 0.001 '**' 0.01 '*' 0.05 '.' 0.1 ' ' 1
```

# We find more sperm in the channel with FRF.

### Is the model OK? should we worry about overdispersion?

```
disp_glmer(mod1)  #not overdispersed
```

```
## [1] 0.9656133
```

```
overdisp_fun(mod1)#not overdispersed
```

```
##       chisq       ratio         rdf           p 
##  38.8234289   0.3156376 123.0000000   1.0000000
```

```
plot((mod1)) #residuals are looking ok
```

```
shapiro.test(residuals(mod1)) #residuals are normally distributed
```

```
## 
##  Shapiro-Wilk normality test
## 
## data:  residuals(mod1)
## W = 0.99386, p-value = 0.8616
```

# The model is OK.

## Checking the effect of SV, TA and sperm pool concentration on the number of sperm collected

```
mod2<-glmer(Y~Time*ul+spermM+(1|Trial)+(1|ID),datachemotaxis2,family="binomial")
#summary(mod2)
Anova(mod2) #effect of fixed effects
```

```
## Analysis of Deviance Table (Type II Wald chisquare tests)
## 
## Response: Y
##          Chisq Df Pr(>Chisq)  
## Time    6.2898  2    0.04307 *
## ul      5.5163  2    0.06341 .
## spermM  0.4859  1    0.48578  
## Time:ul 2.4665  4    0.65064  
## ---
## Signif. codes:  0 '***' 0.001 '**' 0.01 '*' 0.05 '.' 0.1 ' ' 1
```

```
#plot(allEffects(mod2)) # to have quick look a the effects
```

# Sampling time has an effect. Volume doesn’t but it is close. Sperm concentration does not affect the number of sperm collected.

### Is the model OK? should we worry about overdispersion?

```
disp_glmer(mod2) #not overdispersed
```

```
## [1] 0.9687731
```

```
overdisp_fun(mod2) #not overdispersed
```

```
##       chisq       ratio         rdf           p 
##  44.5557919   0.3908403 114.0000000   1.0000000
```

```
plot((mod2))#residuals are looking ok
```

```
shapiro.test(residuals(mod2)) #residuals are normally distributed
```

```
## 
##  Shapiro-Wilk normality test
## 
## data:  residuals(mod2)
## W = 0.99189, p-value = 0.6789
```

The model is OK.

## Testing if pool quality effects the number of sperm collected

### With the combination of TA=20 and SV=3µl

```
corr_data<-filter(datachemotaxis2,Time==20,ul==3)
cor.test(sqrt(corr_data$FRF_n_C),corr_data$VCL)
```

```
## 
##  Pearson's product-moment correlation
## 
## data:  sqrt(corr_data$FRF_n_C) and corr_data$VCL
## t = 1.365, df = 12, p-value = 0.1973
## alternative hypothesis: true correlation is not equal to 0
## 95 percent confidence interval:
##  -0.2035690  0.7510893
## sample estimates:
##       cor 
## 0.3666076
```

```
cor.test(sqrt(corr_data$FRF_n_C),corr_data$LIN)
```

```
## 
##  Pearson's product-moment correlation
## 
## data:  sqrt(corr_data$FRF_n_C) and corr_data$LIN
## t = -0.50947, df = 12, p-value = 0.6197
## alternative hypothesis: true correlation is not equal to 0
## 95 percent confidence interval:
##  -0.6276304  0.4172899
## sample estimates:
##        cor 
## -0.1455053
```

```
cor.test(sqrt(corr_data$FRF_n_C),corr_data$BCF)
```

```
## 
##  Pearson's product-moment correlation
## 
## data:  sqrt(corr_data$FRF_n_C) and corr_data$BCF
## t = -1.0519, df = 12, p-value = 0.3136
## alternative hypothesis: true correlation is not equal to 0
## 95 percent confidence interval:
##  -0.711455  0.283771
## sample estimates:
##        cor 
## -0.2905554
```

```
cor.test(sqrt(corr_data$FRF_n_C),corr_data$MOT)
```

```
## 
##  Pearson's product-moment correlation
## 
## data:  sqrt(corr_data$FRF_n_C) and corr_data$MOT
## t = -0.11715, df = 12, p-value = 0.9087
## alternative hypothesis: true correlation is not equal to 0
## 95 percent confidence interval:
##  -0.5544355  0.5058523
## sample estimates:
##         cor 
## -0.03379859
```

```
cor.test(sqrt(corr_data$FRF_n_C),corr_data$Longev)
```

```
## 
##  Pearson's product-moment correlation
## 
## data:  sqrt(corr_data$FRF_n_C) and corr_data$Longev
## t = 0.26016, df = 12, p-value = 0.7992
## alternative hypothesis: true correlation is not equal to 0
## 95 percent confidence interval:
##  -0.4745449  0.5823311
## sample estimates:
##        cor 
## 0.07489086
```

```
##change the chemotaxis2 to chemotaxis to get the resutls with the entire dataset
```

# No, it doesn’t.

## The code for figure 2:

```
figure2A<-datachemotaxis %>% ggplot(aes(x=Time))+
        geom_boxplot(aes(y=Perc_FRF, fill=ul), col="black", lwd=1)+
        geom_boxplot(aes(y=Perc_H2O, fill=ul),alpha=1, col="grey35",lwd=1)+
        theme_bw()+xlab("")+ylab("")+
        theme(axis.title.x = element_text(size = 16))+
        theme(axis.text.x = element_text(size = 14))+
        theme(axis.title.y = element_text(size = 16))+
        theme(axis.text.y = element_text(size = 14))

figure2B<-datachemotaxis2 %>% ggplot(aes(x=Time))+
        geom_boxplot(aes(y=Perc_FRF, fill=ul), col="black", lwd=1)+
        geom_boxplot(aes(y=Perc_H2O, fill=ul),alpha=1, col="grey35",lwd=1)+
        xlab("")+ylab("")+
        theme_bw()+
        theme(axis.title.x = element_text(size = 16))+
        theme(axis.text.x = element_text(size = 14))+
        theme(axis.title.y = element_text(size = 16))+
        theme(axis.text.y = element_text(size = 14))

Figure2<-ggarrange(figure2A,figure2B, 
          labels = c("A", "B"),
          common.legend = T,
          ncol = 2, nrow = 1)

annotate_figure(Figure2,
                top = text_grob("Sampling Volume (µl)", face = "bold", size = 14),
                left = text_grob("Proportion of Sperm Sampled ± S.E.", size = 16, rot = 90),
                fig.lab = "Figure 1", fig.lab.face = "bold",
                bottom = text_grob("Time After Activation (s)", size = 16))
```

### Calculating averages and SE for figure 3

```
data.summaryS <- datachemotaxis %>%
        group_by(Trial) %>% 
        summarise(
                sd_FRF_N = sd(FRF_n_C, na.rm = TRUE),
                aver_FRF_N = mean(FRF_n_C),
                se_FRF_N= sd_FRF_N/sqrt(n()),
                sd_H_N = sd(H2O_n_C, na.rm = TRUE),
                aver_H_N = mean(H2O_n_C),
                se_H_N= sd_H_N/sqrt(n()),
                poolconc=mean(spermM),
                sd_FRF_Perc = sd(Perc_FRF, na.rm = TRUE),
                aver_FRF_perc = mean(Perc_FRF),
                se_FRF_Perc= sd_FRF_Perc/sqrt(n()),
                sd_H_Perc = sd((Perc_H2O), na.rm = TRUE),
                aver_H_Perc = mean(Perc_H2O),
                se_H_Perc= sd_H_Perc/sqrt(n()))
```

## Figure 3

```
data.summaryS %>% ggplot(aes(x=Trial))+
        geom_pointrange(aes(y=aver_FRF_N,ymin=aver_FRF_N-se_FRF_N,ymax=aver_FRF_N+se_FRF_N),shape=21,color="grey19",fill="grey49",size=1.2)+
        geom_pointrange(aes(y=aver_H_N,ymin=aver_H_N-se_H_N,ymax=aver_H_N+se_H_N),col="black",shape=8,size=1.2)+
        geom_pointrange(aes(y=aver_FRF_perc*100,
                            ymin=aver_FRF_perc*100-se_FRF_Perc*100, 
                            ymax=aver_FRF_perc*100+se_FRF_Perc*100),
                        shape=23,fill="red",color="grey29",size=1.2,alpha=0.8)+
        geom_hline(yintercept=50, linetype="dashed", color = "red",size=2)+
        ylab("Average number 
  of sperm collected ± S.E.")+
        xlab("Trial")+theme_bw()+
        theme(axis.title.x = element_text(size = 16))+
        theme(axis.text.x = element_text(size = 12))+
        theme(axis.title.y = element_text(size = 16))+
        theme(axis.text.y = element_text(size = 12))+
        scale_y_continuous(sec.axis = sec_axis(trans=~.*1, name="Percentage of sperm 
in the FRF ± S.E."))+
        theme(axis.text.y.right = element_text(color = "red"),axis.title.y.right = element_text(color = "red"))
```

## Testing repeatability of the method

### This may take some time

```
data.rep<-read_xlsx("Dataset.xlsx",sheet=2)
data.rep2<-data.rep %>% filter(!(Trial == 5)) #removing a replicate there were in in one case more sperm in the water than in the FRF were found

RN_FRF_Perc<-rpt(Perc_FRF ~ (1 | FRF_id), grname = "FRF_id", data = data.rep,
                 nboot = 1000, npermut = 1000)

RN_FRF_Perc2<-rpt(Perc_FRF ~ (1 | FRF_id), grname = "FRF_id", data = data.rep2, 
                  nboot = 1000, npermut = 1000)
```

```
RN_FRF_Perc #the repeatability with all the trials
```

```
## 
## 
## Repeatability estimation using the lmm method 
## 
## Repeatability for FRF_id
## R  = 0.63
## SE = 0.207
## CI = [0.068, 0.89]
## P  = 0.0178 [LRT]
##      0.008 [Permutation]
```

```
RN_FRF_Perc2 #the repeatability excluding trial N°5
```

```
## 
## 
## Repeatability estimation using the lmm method 
## 
## Repeatability for FRF_id
## R  = 0.878
## SE = 0.106
## CI = [0.573, 0.964]
## P  = 0.000222 [LRT]
##      0.001 [Permutation]
```

```
plot(RN_FRF_Perc) # visualization of the repeatability with C.I. Not shown in the manuscript
```

```
plot(RN_FRF_Perc2) # visualization of the repeatability with C.I., without trial 5. Not shown in the mansucript
```

# The method is repeatable

### Figure S4

```
data.rep %>% ggplot(aes(x=as.factor(Trial))) +
        geom_point(aes(y=Perc_FRF),colour="black",size=6,fill=NA,stroke=1)+ 
        geom_point(aes(y=Perc_FRF,color=as.factor(Sperm_Pool)),size=5)+
        xlab("Trial Number")+ylab("Proportion of Sperm Sampled in FRF")+
        labs(col= "Sperm Pool")+theme_bw()+
        theme(axis.title.x = element_text(size = 16))+
        theme(axis.text.x = element_text(size = 12))+
        theme(axis.title.y = element_text(size = 16))+
        theme(axis.text.y = element_text(size = 12))+
        theme(legend.text = element_text(size = 14))+
        theme(legend.title = element_text(size = 16))
```
